# Evaluation of the equation for predicting dry matter intake of lactating dairy cows in the Korean feeding standards for dairy cattle

**DOI:** 10.5713/ajas.20.0684

**Published:** 2020-11-09

**Authors:** Mingyung Lee, Junsung Lee, Seoyoung Jeon, Seong-Min Park, Kwang-Seok Ki, Seongwon Seo

**Affiliations:** 1 Division of Animal and Dairy Sciences, Chungnam National University, Daejeon 34134, Korea; 2 National Institute of Animal Science, Rural Development Administration, Cheonan 31000, Korea

**Keywords:** Dry Matter Intake, Lactating Dairy Cow, Meta-analysis, Modeling

## Abstract

**Objective:**

This study aimed to validate and evaluate the dry matter (DM) intake prediction model of the Korean feeding standards for dairy cattle (KFSD).

**Methods:**

The KFSD DM intake (DMI) model was developed using a database containing the data from the Journal of Dairy Science from 2006 to 2011 (1,065 observations 287 studies). The development (458 observations from 103 studies) and evaluation databases (168 observations from 74 studies) were constructed from the database. The body weight (kg; BW), metabolic BW (BW^0.75^, MBW), 4% fat-corrected milk (FCM), forage as a percentage of dietary DM, and the dietary content of nutrients (% DM) were chosen as possible explanatory variables. A random coefficient model with the study as a random variable and a linear model without the random effect was used to select model variables and estimate parameters, respectively, during the model development. The best-fit equation was compared to published equations, and sensitivity analysis of the prediction equation was conducted. The KFSD model was also evaluated using *in vivo* feeding trial data.

**Results:**

The KFSD DMI equation is 4.103 (±2.994)+0.112 (±0.022)×MBW+0.284 (±0.020) ×FCM−0.119 (±0.028)×neutral detergent fiber (NDF), explaining 47% of the variation in the evaluation dataset with no mean nor slope bias (p>0.05). The root mean square prediction error was 2.70 kg/d, best among the tested equations. The sensitivity analysis showed that the model is the most sensitive to FCM, followed by MBW and NDF. With the *in vivo* data, the KFSD equation showed slightly higher precision (R^2^ = 0.39) than the NRC equation (R^2^ = 0.37), with a mean bias of 1.19 kg and no slope bias (p>0.05).

**Conclusion:**

The KFSD DMI model is suitable for predicting the DMI of lactating dairy cows in practical situations in Korea.

## INTRODUCTION

Dry matter intake (DMI) is a major factor determining milk production in lactating dairy cows. Accurate estimation of the DMI is thus important to diet formulation to prevent under- or over-feeding, which is directly related to the health and performance of cows [1] and farm income [2]. Several models for predicting DMI have been proposed worldwide, and similarly, in South Korea, researchers have sought to develop a DMI model that can be used in domestic environments.

The first edition [3] and the first revised edition [4] of the Korean feeding standards for dairy cattle (KFSD) adopted the empirical model presented by NRC [1], which is the most widely used DMI prediction equation for lactating dairy cows. For the second revised edition of the KFSD, the program committee considered developing a new DMI model since the NRC [1] model was based on experimental data from the early 1990s. When evaluated with the latest data, the NRC equation (and other existing DMI equations) showed significant mean and slope biases [5]. Therefore, the second revision of the KFSD included a new empirical DMI prediction model for lactating dairy cows using data from 2006 to 2011 [6]. However, the adequacy of development procedure of the model was not validated. Moreover, the model was not evaluated with *in vivo* feeding trial data conducted in Korea.

The objectives of this study were i) to describe the development of the DMI prediction model of the KFSD, ii) to evaluate and compare the predictability of the model with several existing DMI equations, and iii) to evaluate the model using *in vivo* feeding data conducted in a Korean dairy farm.

## MATERIALS AND METHODS

### Database construction

A literature database based on the research articles published in the Journal of Dairy Science from January 2006 to June 2011 (volumes 89 to 94) was constructed and contained 1,065 treatment means from 287 studies (Table 1). Data were excluded if i) the milk yield (MY) was not reported, ii) the breed was other than Holstein, iii) the DMI as a percentage of body weight (DMIpBW) was less than 2%, iv) the forage as a percentage of dietary dry matter (FpDM) was less than 30% or 100%, v) the dietary neutral detergent fiber (NDF) content in the dry matter (DM) basis was less than 25%, or vi) the week of lactation was missing. Among the studies that met the above criteria, those containing less than four observed means were assigned to the evaluation dataset - four observations were selected to balance the required sample size to estimate the correlations among variables within each study and the number of studies used for developing an equation [7]. The final dataset for developing a DMI equation included 458 observations from 103 studies (Table 2).

### Model development

The variables that can be routinely measured or calculated in practice were selected as the candidate explanatory variables of DMI (kg/d). These included the animal factors (i.e., body weight [BW, kg], metabolic BW [MBW, kg^0.75^]), milk production (i.e., MY [kg/d]), and 4% fat-corrected milk yield [FCM, kg/d]) and the dietary components and their intake (i.e., FpDM [%], NDF [% DM], crude protein [CP, % DM], starch [% DM], acid detergent fiber [% DM], CP intake [kg/d], NDF intake [kg/d], starch intake [kg/d], and forage intake [kg/d]). Unlike the 2001 dairy NRC [1], the dietary components were also included because they may limit the intake of lactating dairy cows [8].

The development procedure was divided into two phases. The first phase was to identify the independent variables that were statistically significant for DMI prediction. A random coefficient model with study as the random variable was used via the MIXED procedure in SAS (SAS Institute, Carey, NC, USA). The statistical model in matrix notation is:

y=Xβ+Zu+e

where *y* is the vector of the observed DMI with size *N*, *X* is the *n*×*p* matrix of *x_i,j_*, *β* is the parameter vector with size *p* for the fixed effects, *Z* is the designed *N*×(*s*×*p*) matrix that was blocked diagonally corresponding to each study (*n_i_*×p) to account for the random effect of study, *N* is the total number of observations, *s* is the number of studies, *n_i_* is the number of observations that study *i* contained, *p* is the number of parameters, which is one (intercept) plus the number of variables used in the equation, *e* and *u* are the vectors of independent, identically and normally distributed random errors (*E*[*u*] = 0, *Var*[*u*] = *G*, *E*[*e*] = 0, *Var*[*e*] = *R*), *G* is the (*s*×*p*)×(*s*×*p*) matrix that contained the variance components in a diagonal structure with a block matrix for each study, and *R* is σ^2^*I_N_* when *I_N_* denotes the *N*×*N* identity matrix. Among the acceptable regression models with a linear combination of the significant explanatory variables, the model with the lowest Akaike’s (AIC) and Bayesian information criteria (BIC) was selected.

In the second phase, the parameters of the variables in the best model from phase one were estimated by fitting the model via the general linear model procedure in SAS. Reduced DMI in the early stages of lactation was accounted for by using the equation of Roseler et al [2], who developed a lag function for adjusting the DMI during the first 16 weeks of lactation. The lag function is an exponential function of the current week of lactation and a different value for the month of lactation when peak MY occurs. The Lag[2] assumes peak MY occurs in the second month of lactation and uses a value of 2.36, while Lag[3] uses a value of 3.67 and assumes that peak MY occurs in the third month of lactation. The value calculated by the lag function is a multiplier to the basic DMI equation. The NRC and the Cornell Net Carbohydrate and Protein System (CNCPS) use this function, but with different assumptions for the month of lactation when peak MY occurs. NRC uses Lag[3], while CNCPS uses Lag[2]. The equation developed in this study uses Lag[2]; the peak MY occurred in the second month of lactation in Korea [9]. The lag function used in our model is:

$$\text{Lag}[2]=1-{exp}^{\left(-0.318\times (week\;of\;lactation+2.36)\right)}$$

### Evaluation and comparison of the KFSD DMI equation

The KFSD equation developed in this study was compared to other published DMI equations using the evaluation dataset. The data in the evaluation dataset did not contain the information for any of the input variables of the tested equations, it was excluded from the evaluation database; the final evaluation database included 168 observations from 74 studies (Table 2). The KFSD DMI equation was compared to the NRC [1], CNCPS [10], and the Japanese feeding standard (JFS) [11] model (Table 3). To evaluate the NRC and CNCPS equations, the base equations with different lag equations were also examined (i.e., NRC with Lag[2], NRC with Lag[3], CNCP with Lag[2], and CNCPS with Lag[3]). The JFS DMI equation was included because it is one of the most recently developed empirical equations. Other previously published DMI equations [2,12–14] were not included because initial comparisons indicated that the NRC and CNCPS models were superior [5]. The coefficient of determination (R^2^) and root mean square prediction error (RMSPE) were used as indicators of the model precision and accuracy, respectively. Residual analyses were also conducted to assess the mean and slope biases of the models.

### Sensitivity analysis

A sensitivity analysis of the DMI prediction equation to input variables was conducted in @Risk version 7.0 (Palisade Corp., Newfield, NY, USA). The distribution of the input variables was generated from a data set of 629 observations of lactating dairy cows from the development and evaluation datasets. Probability distributions were fit to the data for each variable in @Risk, and the best distribution was selected by comparing three different fit statistics (χ^2^, Kolmogorov-Smirnov, and Anderson-Darling values). The data were assumed to be normally distributed if all three goodness-of-fit tests failed to reject the null hypothesis; otherwise, a probability distribution, which showed the best fit among the three tests (based on ranking), was chosen. Simulations were performed to obtain the distribution of the predicted DMI in @Risk, and the input variables needed to predict DMI were sampled from each distribution using the Latin hypercube method. The iterations of the simulation were continued until <1% convergence was achieved for a DMI distribution. The sensitivity of the DMI equation to the input variables was analyzed by regression analysis in @Risk, and the coefficients from a standardized regression were used to rank the relative importance of the input variables.

### Evaluation of the KFSD DMI equation with *in vivo* feeding trials

The predictive power and accuracy of the KFSD DMI equation for lactating dairy cows were evaluated using *in vivo* experimental data. The data were collected from November 2016 to June 2017 from a research dairy farm for the National Institute of Animal Science in Chungcheong Province, Republic of Korea. All animal usage and experimental procedures were conducted with the approval of the Institutional Animal Care and Use Committee at the National Institute of Animal Science, Rural Development Administration, Republic of Korea (Approval number: 2016-173) in 2016. The study used 32 Holstein lactating cows (746±75.1 kg), and the average parity of the cows was 2.1±1.00 (11 primiparous and 21 multiparous). The cows were housed together in a bedded pack barn with wood shavings, equipped with an automatic milking system (Astronaut 3, Lely, Wageningen, Netherlands). The cows were fed total mixed rations (TMR) *ad libitum*, provided twice daily at 09:00 and 17:00, and their individual daily intakes were recorded by an automatic feeding system with radio frequency identification (Dawoon Co., Incheon, Korea). The intake and MY data were collected from the automatic milking system, and the nutrient composition of the TMR and the concentrate mix are described in Table 4. Feed samples were analyzed for proximate constituents and fiber fractions as per AOAC [15] and Van Soest et al [16], respectively. Milk samples were collected from each cow once a week (in triplicate) to analyze the fat and protein contents via infrared spectroscopy (MilkoScan 4000; Foss Electric, Hillerød, Denmark).

The entire data set contained individual intake and milk production data for seven months, but the data for February was removed due to problems with data collection. The average and standard deviation for each individual and parameter was calculated; observations greater or less than three times the standard deviation were excluded as outliers. The weekly and monthly averages for each individual and parameter were also calculated, and the database included 673 weekly and 173 monthly observations. The descriptive statistics for the monthly observations are provided in Table 5, and this dataset was used to evaluate the model. Model evaluation was also performed by categorizing the data set into cold (November to January) and warm (March to June) seasons. The model was compared to the NRC (2001) equation [1], which has shown higher predictive power than the other published models. R^2^ and RMSPE were used as indicators of model precision and accuracy, respectively, and residual analyses were conducted to assess the mean and slope biases of the models.

## RESULTS AND DISCUSSION

### Development of the KFSD DMI equation

In the first phase, a linear combination of MBW, FCM, and NDF was the best-fitting model among all of the possible linear combinations of the candidate variables (data not shown). As indicated by the values (means and standard deviations) in Table 2, these equations cover a wide range of production situations. The parameters of MBW, FCM, and NDF were estimated using ordinary least squares as follows:

DMI=4.103(±2.994)+0.112(±0.022)×MBW+0.284(±0.020)×FCM-0.119(±0.028)×NDF

where MBW is the metabolic body weight (kg, BW^0.75^), FCM is the 4% fat-corrected milk (kg/d), and NDF is the NDF content in the dietary DM (%DM).

The nutritional requirements are fundamentally determined by body weight [17], so many DMI prediction models have included BW [5,10,18,19]. MBW, defined as the three-fourth power of body weight, has also been frequently used in previously published models [1,11,13]. In this study, MBW was a more appropriate variable to predict DMI than BW (data not shown). Other studies have also found that models including MBW exhibit better accuracy and lower prediction error than models including BW [20]. Feed intake is determined by the energy requirements [21], which, in lactating dairy cows, can be divided into the requirements for maintenance and milk production. The maintenance requirements are proportional to the MBW, indicating the amount of active tissue or metabolic mass [22], so MBW is more likely to act as a reliable variable in the DMI prediction model.

The feed intake of lactating cows is positively correlated with milk production [23], suggesting that increased milk production leads to an increase in DMI. Thus, several prior studies have used MY or FCM as an input variable in the DMI prediction model for lactating cows. The results of this study show that FCM is a more suitable variable to predict DMI than MY (data not shown). FCM with MY adjusted to 4% fat was consistently included in models more often than MY [6,11,12,18,24], and the energy required for lactation is proportional to FCM [25]. Therefore, FCM may improve the predictive power of the DMI prediction model.

The only dietary factor included in our final equation was NDF. The NDF content of the feed is a reliable predictor of DMI due to its strong correlation with DMI [26]; there is a positive correlation between NDF and rumen fill, and a negative correlation between NDF and energy density [18]. NDF has low digestibility and negatively affects DMI by increasing the rumen residence time and physical satiety. Previous studies found that when the NDF content of the feed was more than 25%, the intake reduced due to rumen filling, but when the NDF content was less than 25%, the intake decreased due to metabolic feedback [2,8,19]. Feed intake is also affected by the bulk density, digestion rate, time spent ruminating and chewing, and the disappearance rate of the feed [27], all of which are strongly correlated with the NDF content. Thus, NDF has often been included in the DMI prediction equations [13,14,28].

### Evaluation of the KFSD DMI equation with an independent dataset

Table 6 summarizes the statistics from the regression analyses of the KFSD DMI equation and the previously published models when evaluated with the evaluation data set. The precision of the models (R^2^) was similar (0.46 to 0.48), except for the JFS model (R^2^ = 0.44). The R^2^ value of the regression of the observed and predicted values is widely used as a precision index when the relationship is linear. The RMSPE value represents the average vertical distance of each point to the predicted value in a residual plot and measures how well the predictions fit the observed data. It is a common, reliable estimate of the predictive accuracy of a model. The KFSD equation had the lowest RMSPE value (2.7 kg) among the previously published models (Table 6), and the KFSD DMI predictive model reduced RMSPE by a minimum of 0.1 and a maximum of 0.98 compared to other models.

**Figure 1:**
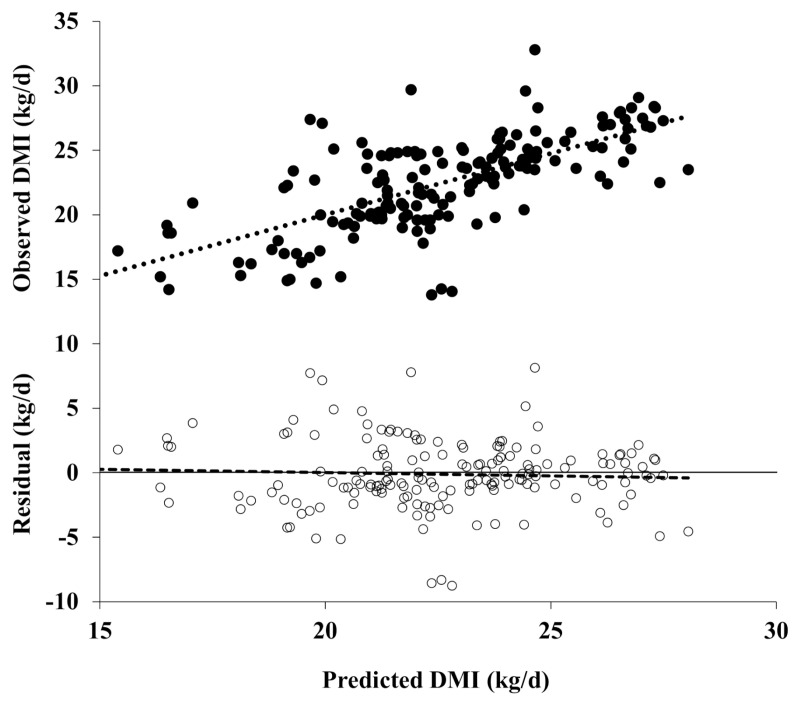
Plots of the observed (upper; ●) and residual DMI (observed minus predicted DMI, lower; ○) versus the model-predicted DMI (n = 168). The regression equation was DMI = 0.95×predicted DMI+1.04 (R^2^ = 0.47, RMSPE = 2.70 kg/d). For the residual analysis, the predicted DMI was centered around the mean predicted DMI before the observed minus predicted (residual) DMI were regressed on the predicted values. The regression equation was residual DMI = −0.122(±0.2091)−0.051(±0.0781)×(predicted DMI−22.5480). DMI, dry matter intake; RMSPE, root mean square prediction error.

Figure 1 shows plots of the observed and residual DMI values (observed minus predicted DMI) versus the predicted DMI values from a model (using the evaluation dataset). When the residual values are regressed on the predicted values that are centered on the mean predicted value, the regression slope and intercepts represent linear and mean biases, respectively [29]. As shown in Table 6 and Figure 1, the intercept and slope of the regression did not differ from 0, indicating that the KFSD equation is unbiased (p>0.05). However, the other models showed statistically significant mean biases (−0.563 to 2.347, p<0.01), except for the NRC (2001) equation. The slope biases were statistically significant, except for the CNCPS model (−0.065 to −0.203, p<0.05). Therefore, the precision, accuracy, and biases assessments suggest that the KFSD DMI prediction model is better than the other tested models.

**Figure 2:**
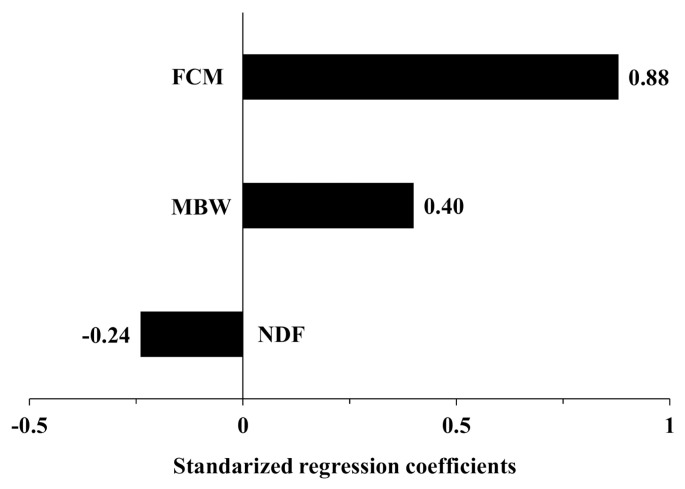
Tornado plot of the standardized regression coefficients for the model inputs. The inputs were ranked based on their standard regression coefficient as the most influential in predicting dry matter intake of the lactating dairy cattle with the Korean feeding standards for dairy cattle (KFSD) equation. FCM is the 4% fat-corrected milk yield (kg), MBW is the metabolic body weight (kg^0.75^), and NDF is the dietary neutral detergent fiber content (% DM).

The tornado graphs in Figure 2 show the sensitivities of the predicted DMI outputs to the input variables. The standardized regression coefficients indicate the relative importance of the variables and suggest that a one-unit increase in the variable would increase the DMI by one unit (i.e., kg). In the developed KFSD model, the regression coefficient of FCM (0.9) was positive and twice that of the other variables. NDF, the only variable that was negatively correlated with DMI, had a regression coefficient of −0.2, indicating that a 1% p increase in dietary NDF (%) would decrease 0.2 kg of DMI.

### Evaluation of the KFSD DMI equation using data from Korean dairy farms

**Figure 3:**
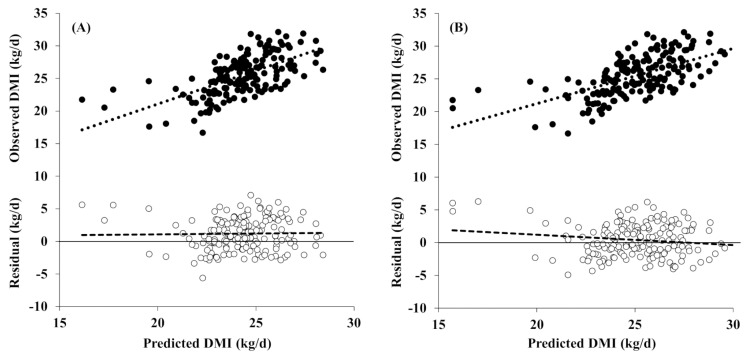
Plots of the observed (upper; ●) and residual DMI (observed minus predicted DMI, lower; ○) versus the model-predicted DMI of (A) the KFSD equation and (B) the NRC equation (n = 173). The regression equations were: (A) observed DMI = 1.03×predicted DMI+0.52 (R^2^ = 0.39) and (B) observed DMI = 0.84×predicted DMI+4.35 (R^2^ = 0.37). For the residual analysis, the predicted DMI was centered around the mean predicted DMI before the observed minus predicted (residual) DMI were regressed on the predicted values. The regression equations were: (A) residual DMI = 1.187(±0.1866)+0.028(±0.0993)×(predicted DMI−24.2674) and (B) residual DMI = 0.414(±0.1889)−0.157(±0.0841)×(predicted DMI−25.0406).

The evaluation results for the KFSD model and the NRC model using *in vivo* experimental data from a Korean dairy farm are shown in Figure 3 and Table 7. The KFSD model had slightly lower accuracy (RMSPE = 2.71) than the NRC model, but slightly higher precision (R^2^ = 0.39). The KFSD prediction model had no slope bias (0.03; p>0.05), but it had a significant mean bias (1.2 kg; p<0.01). The NRC model had a significant mean bias (0.41 kg; p<0.05), and the slope tended to bias (−0.16; p = 0.06).

The significant mean bias of the KFSD equation may be because it underpredicts the DMI of lactating cows during the cold season. We reanalyzed the predictive power of the KFSD equation using data from the cold season (November to January); R^2^ and RMSPE were 0.46 and 3.1, respectively, with no slope bias (p>0.05) but a significant (p<0.001) mean bias (2.34 kg) (Table 7). In contrast, neither the mean nor the slope biases were significant when evaluated with the warm season data (March to June), and the R^2^ and RMSPE values were 0.37 and 2.43, respectively. With the warm season data, the NRC (2001) equation was not significantly biased (similar to the KFSD equation), but with the cold season data, both the mean and slope were significantly biased (p< 0.05). These results indicate a structural error in predicting the DMI of lactating cows in Korea during the cold season.

The KFSD equation predicted a DMI of ~2.3 kg less than the actual value in the cold season because it seems to have not considered the effect of temperature. In general, when the temperature decreases, the feed intake increases to compensate for the increased energy requirements of maintaining body temperature [30]. The KFSD equation corrects the intake according to the environmental temperature (not proposed in this paper), but this was not considered here because the experimental data lacked information on temperature, humidity, and wind speed. The equation including the environmental module predicts a DMI increase of 9.7% in lactating cows when the monthly average temperature is −10°C (data not shown). The average intake in the evaluation data set was 25.3 kg, and the increased DMI at −10°C was ~2.3 kg, which is similar to the mean bias calculated for the winter data.

In conclusion, the KFSD DMI prediction equation can predict the DMI of lactating cows in Korea with relatively good precision (R^2^ = 0.35 to 0.47; no structural biases were observed). The KFSD equation has a relatively high predictive power compared to the other previously published equations, including the prediction equation of the NRC (2001) [1]. However, the KFSD equation may underpredict DMI in the cold season by approximately 2.3 kg. If the DMI increase at low temperatures is evaluated with the environmental module, then the mean bias may be significantly reduced.

## Figures and Tables

**Table 1:** Descriptive statistics of the animal information, milk yield and composition, and the dietary nutrient composition of the data in the literature database

Variables	n	Mean	SD	Median	Max	Min
Animal						
BW (kg)	1,065	615.97	109.65	644.00	820.00	40.00
DMI (kg/d)	1,065	20.52	5.64	21.80	32.80	0.58
DMIpBW (% BW)	1,065	3.31	0.72	3.48	5.04	1.04
Milk composition						
MY (kg)	933	33.89	6.68	34.30	53.80	13.40
4% FCM (kg)	897	31.90	5.99	32.22	48.20	14.97
Milk fat (%)	897	3.60	0.50	3.60	5.93	1.83
Milk protein (%)	853	3.13	0.31	3.09	6.87	2.53
Dietary nutrient composition						
FpDM (% DM)	1,051	53.35	12.82	51.00	100.00	19.70
DM (% as fed)	728	55.94	12.37	54.10	96.20	17.15
CP (% DM)	1,037	16.83	2.35	16.90	28.15	7.10
EE (% DM)	509	4.43	2.50	3.90	19.70	1.50
NDF (% DM)	1,012	34.31	6.35	33.60	65.90	11.30
ADF (% DM)	772	21.07	4.77	20.45	43.80	4.80
Starch (% DM)	404	24.31	6.28	24.20	42.30	5.20

SD, standard deviation; BW, body weight; DMI, dry matter intake; DMIpBW, dry matter intake as a percentage of body weight; MY, milk yield; FCM, fat-corrected milk; FpDM, forage as a percentage of the dietary dry matter; CP, crude protein; EE, ether extract; NDF, neutral detergent fiber; ADF, acid detergent fiber.

**Table 2:** Descriptive statistics of the development and evaluation databases

Variables	Development				Evaluation			
	
	n	Mean	SD	Median	n	Mean	SD	Median
BW (kg)	458	640.33	50.29	638.65	168	647.17	49.31	644.00
DMI (kg)	458	22.66	3.18	22.70	168	22.46	3.69	22.90
MY (kg)	458	34.43	5.81	35.10	168	34.71	7.78	34.40
4% FCM kg	458	31.22	6.36	31.97	168	32.23	7.91	32.96
Milk fat (%)	443	3.49	0.46	3.50	165	3.65	0.44	3.63
Milk protein (%)	416	3.11	0.24	3.09	149	3.09	0.25	3.09
Forage DMI (kg)	458	11.52	2.24	11.48	168	11.31	2.30	11.15
NDF (% DM)	444	33.35	4.51	32.80	168	33.54	4.31	33.54
ADF (% DM)	357	20.27	3.39	19.80	120	19.98	2.97	20.10
CP (% DM)	444	16.93	1.76	16.95	168	17.19	2.17	17.30

SD, standard deviation; BW, body weight; DMI, dry matter intake; MY, milk yield; FCM, fat-corrected milk; NDF, neutral detergent fiber; ADF, acid detergent fiber; CP, crude protein.

**Table 3:** Published prediction equations of the dry matter intake (DMI, kg/d) of lactating dairy cows compared to the developed KFSD DMI equation

Model^1)^	Equation^2)^
NRC Lag[x]^3)^	(0.372×FCM+0.0968×BW^0.75^)×Lag[x]
CNCPS Lag[x]^3)^	(0.0185×BW+0.305×FCM)×Lag[x]
JFS	Multiparous 1.3922+0.05839×BW^0.75^+0.40497×FCM×[1.0−0.3531×exp(−0.3247×WOL)] Primiparous 1.9120+0.07031×BW^0.75^+0.34923×FCM×[1.3671−0.6558×exp(−0.0498×WOL)]

1) NRC, National Research Council [1]; CNCPS, Cornell Net Carbohydrate and Protein System [10]; JFS, Japanese feeding standard for dairy cattle [11].

2) FCM, 4% fat-corrected milk; BW, body weight; WOL, week of lactation

3) Lag[x] is the lag function for adjusting the DMI reduction during the first 16 weeks of lactation when the peak milk yield occurs on the x month of lactation [2]. Lag[2] = 1−exp(−0.316×[WOL+2.36]), Lag[3] = 1−exp(−0.192×[WOL+3.67]).

**Table 4:** Nutrient composition (g/kg DM or as stated) of the total mixed ration and concentrate mix used in the feeding trial with lactating Holstein cows

Nutrients	Total mixed ration	Concentrate mix
DM (g/kg as fed)	510	883
CP	125	217
EE	47	48
CF	224	89
Ash	65	77
NDF	432	288
ADF	245	143
Ca	7	11
P	4	6
TDN	698	778
NEl (MJ/kg DM)	6.7	7.5

DM, dry matter; CP, crude protein; EE, ether extract; CF, crude fiber; Ash, crude ash; NDF, neutral detergent fiber; ADF, acidic detergent fiber; Ca, calcium; P, phosphorus; TDN, total mixed ration; NEl, net energy for lactation.

**Table 5:** Descriptive statistics of the feeding trial data used to evaluate the DMI prediction equation

Variables	Mean	SD	Median	Max	Min
BW (kg)	751	79.5	741	991	601
DMI (kg)	25.3	3.06	25.3	32.1	16.7
MY (kg)	31.2	7.13	31.9	50.2	3.6
Milk fat (%)	4.2	0.63	4.1	6.3	2.4
Milk protein (%)	3.5	0.34	3.5	4.6	2.6
4% FCM (kg)	31.9	6.47	32.1	50.2	3.4
Dietary NDF (% DM)	40.0	0.98	39.9	42.8	38.2
TMR DMI (kg)	19.9	2.87	19.9	26.7	12.1
Concentrate DMI (kg)	5.3	1.29	5.7	7.5	1.5

DMI, dry matter intake; SD, standard deviation; BW, body weight; MY, milk yield; FCM, fat-corrected milk; NDF, neutral detergent fiber; TMR, total mixed ration.

**Table 6:** The predictive precision and accuracy of the KFSD DMI equation and other published equations

Model^1)^	Linear regression				Bias			
	
	Intercept	Slope	R^2^	RMSPE^2)^	Mean	p-value	Slope	p-value
KFSD	1.04	0.95	0.47	2.70	−0.122	0.56	−0.051	0.51
NRC (2001)	5.32	0.77	0.46	2.83	0.236	0.23	−0.229	<0.01
CNCPS	2.99	0.94	0.48	3.13	1.629	<0.01	−0.065	0.39
NRC Lag[2]^3)^	3.51	0.82	0.48	2.80	−0.563	<0.01	−0.177	<0.01
CNCPS Lag[3]^3)^	5.26	0.86	0.46	3.62	2.347	<0.01	−0.145	<0.05
JFS	5.52	0.8	0.44	3.09	1.206	<0.01	−0.203	<0.01

1) KFSD, equation developed in this study; NRC, National Research Council [1]; CNCPS, Cornell Net Carbohydrate and Protein System [10]; JFS, Japanese feeding standard for dairy cattle [11].

2) RMSPE, root mean square prediction error

3) Lag[x] is the lag function for adjusting the DMI reduction during the first 16 weeks of lactation when the peak milk yield occurs on the x month of lactation [2]. Lag[2] = 1−exp(−0.316×[WOL+2.36]), Lag[3] = 1−exp(−0.192×[WOL+3.67]). WOL, week of lactation.

**Table 7:** The predictive precision and accuracy of the KFSD DMI and NRC (2001) equations

Model^1),2)^	Linear regression				Bias			
	
	Intercept	Slope	R^2^	RMSPE^3)^	Mean	p-value	Slope	p-value
Overall								
KFSD	0.52	1.03	0.39	2.71	1.19	<0.01	0.03	0.78
NRC (2001)	4.35	0.84	0.37	2.53	0.41	0.03	−0.16	0.06
Cold season								
KFSD	3.80	0.94	0.46	3.12	2.34	<0.01	−0.06	0.63
NRC (2001)	7.99	0.75	0.45	2.71	1.61	<0.01	−0.25	0.02
Warm season								
KFSD	−0.32	1.03	0.37	2.43	0.46	0.05	0.03	0.81
NRC (2001)	2.30	0.89	0.37	2.40	−0.34	0.15	−0.11	0.35

1) Data from March to June except for February (n = 173); Cold season, data from November to January (n = 67); Warm season, data from March to June (n = 106).

2) KFSD, equation developed in this study; NRC, National Research Council [1].

3) RMSPE, root mean square prediction error.
